# Analysis of Volatile Metabolome and Transcriptome in Sweet Basil Under Drought Stress

**DOI:** 10.3390/cimb47020117

**Published:** 2025-02-11

**Authors:** Yuan Zhou, Guangying Ma, Wenlue Li, Lupeng Xie, Shuxia Zhan, Xingda Yao, Ziwei Zuo, Danqing Tian

**Affiliations:** Zhejiang Institute of Landscape Plants and Flowers, Hangzhou 311251, China

**Keywords:** *Ocimum basilicum*, drought stress, volatile metabolome, transcriptome, phenylpropanoids, α-linolenic acid

## Abstract

Basil, renowned for its aromatic properties, exhibits commendable drought tolerance and holds significant value as an edible and medicinal plant. Recognizing the scarcity of studies addressing basil’s response to drought stress, we performed physiological experiments and omics analyses of sweet basil across four distinct levels of drought stress. During drought stress, basil showed increased activity of antioxidant enzymes and accumulated more osmoregulatory compounds. Our metabolic analysis meticulously identified a total of 830 metabolites, among which, 215 were differentially accumulated. The differentially accumulated metabolites under drought stress were predominantly esters and terpenes; however, none were identified as the primary volatile compounds of basil. Transcriptome analyses highlighted the pivotal roles of phenylpropanoid and flavonoid biosynthesis and lipid metabolism in fortifying the resistance of sweet basil against drought stress. α-linolenic acid, lignin, flavonoid, and flavonol contents significantly increased under stress; the essential genes involved in the production of these compounds were confirmed through quantitative real-time PCR (qRT-PCR), and their variations aligned with the outcomes from sequencing. This holistic approach not only enriches our understanding of the molecular intricacies underpinning basil’s drought resistance but also furnishes valuable insights for the molecular breeding of basil varieties endowed with enhanced drought tolerance.

## 1. Introduction

Drought stress remains a persistent and escalating challenge in agriculture [1], contributing to reduced crop yields and substantial global economic losses [2]. The increasing threat of long-term and frequent droughts due to global warming underscores the urgency of addressing drought stress [3], particularly through the breeding of drought-tolerant plants.

The detrimental effects of drought stress on plant growth and development, potentially leading to plant death, necessitate a comprehensive understanding of the morphological, physiological, and molecular transformations that plants undergo in response to increasing aridity [4,5]. Notably, the traditional wheat variety exhibited improved drought tolerance by significantly increasing lateral root length and the length/density of root hairs [6]. Additionally, Tibetan wild barley genotypes, a valuable genetic resource, provide essential genes for enhancing K metabolism and augmenting drought tolerance [7].

The responses of diverse plant species to drought stress are complex and varied, involving the activation of specific drought-related genes and signaling pathways. Abscisic acid (ABA) emerges as a key player in plant drought response, regulating physiological strategies in stress signaling pathways [8]. For instance, the osmotin-like protein, OsOLP1, modulates drought stress by influencing the accumulation of ABA, proline, and lignin [9]. Stomatal density also affects drought tolerance, with rice plants exhibiting reduced stomatal density and demonstrating enhanced drought tolerance [10,11]. Chromatin modifications, such as DNA methylation and ubiquitination, exert significant impacts on plant responses to abiotic stresses [12]. The H3K36 methyltransferase SDG708 enhances rice drought tolerance by promoting abscisic acid (ABA) biosynthesis and reducing water loss [13].

Advancements in omics technologies facilitate a deeper understanding of plant responses to drought stress, unveiling a plethora of metabolites and metabolic pathways. Plant-specific signal pathways responding to drought stress feature distinctive characteristics. Flavonoids, as polyphenolic secondary metabolic compounds, play crucial roles in stress management. Metabolic pathways involving fatty acids, phenylalanine, and flavonoids are identified as crucial mechanisms employed by plants like the Lanzhou lily and soybean to cope with drought stress [14]. Flavonoid levels of *Zanthoxylum* species are altered in response to drought stress [15], as ABA and flavonoid biosyntheses operate as key functional participants in soybean responses to drought [16]. In sesame, amino acid metabolism and ABA metabolism emerge as pivotal drought tolerance signal pathways [17]. Medicago ruthenica adapts to drought by regulating its osmoregulatory and photosynthetic systems, with the ABA and auxin signaling pathways playing critical roles in this adaptation [18]. These findings emphasize the complexity of plant responses to drought stress and highlight potential avenues for developing drought-tolerant varieties.

Basil (*Ocimum basilicum* L.), an annual herb plant belonging to the *Lamiaceae* family, has gained prominence due to its versatility as a widely cultivated aromatic plant. Basil leaves are integral to Western cuisine [19] and boast pharmaceutical properties [20,21]. Abundant in volatile oils such as linalool, eucalyptol, and eugenol [22], basil essential oil exhibits calming and soothing effects on the nervous system, along with anti-inflammatory, antimicrobial, and insect-repelling activities [23,24,25].

Just as with other crops, the productivity and quality of basil are significantly influenced by abiotic stresses, such as salinity and heavy metal exposure. In one study, plant growth was impeded under conditions of salt stress, whereas the concentrations of essential oil, malondialdehyde (MDA), and proline were elevated [26]. Furthermore, the levels of total flavonoids, total phenolic compounds, and total phenolic acids exhibited a significant increase under salt stress conditions. The basil variety characterized by a higher concentration of antioxidant compounds and elevated constitutive levels of proline demonstrated superior adaptation to saline environments [27]. In response to cadmium stress, basil modulates its phytochemical and physiological characteristics, including an increase in the activity of antioxidant enzymes and the concentration of phenolics and flavonoids [28]. Originating from tropical Asia, basil thrives in warm and arid environments, with temperature and water stress posing significant challenges to its growth [29]. Under drought stress conditions, the yield of basil leaves was reduced, while the levels of proline, soluble sugars, total flavonoids, and total phenols increased [30]. Enhancing photosynthesis and the secondary metabolism of phenols and anthocyanins can alleviate the effects of drought stress in purple basil [31]. While plants respond to drought stress by modulating gene expression and metabolite content [32,33], comprehensive omics studies on basil under drought conditions are limited. Although de novo transcriptome sequencing of basil leaves under various stresses has been conducted [34], the molecular mechanisms of drought tolerance remain elusive.

To gain deeper insights into the impact of drought stress on sweet basil and unravel the associated molecular mechanisms, we conducted an experiment using sweet basil as the experimental material. Potted seedlings were subjected to drought stress by withholding water, and their leaves were subsequently analyzed both transcriptomically and metabolomically. The outcomes of this study were anticipated to unveil drought-responsive genes and metabolites, providing theoretical support for molecular breeding and cultivation practices aimed at enhancing basil’s drought tolerance.

## 2. Materials and Methods

### 2.1. Plants and Drought Treatment

The experimental material comprised sweet basil. The cultivation process entailed the initial sowing of seeds onto trays containing 32 individual cells. Two weeks post-germination, the seedlings were subsequently transplanted into pots with a diameter of 12 cm, with each pot accommodating a single seedling. The nutrient-rich soil used for cultivation included a mixture of clay at a 3:2 ratio. The seedlings were nurtured in an artificial climate chamber, maintaining a light intensity of 12,000 lux at 25 °C from 7:00 to 19:00 daily, followed by darkness at 23 °C for the remaining hours. Pinching out the tops of the plants occurred when six true leaves appeared. To initiate the drought treatment, regular watering of the soil continued until the plants reached approximately 11 cm in length. Soil water content (SWC) was monitored using a moisture detector (Horde Electronic Soil Moisture Detector, Shangdong, China). The maximum SWC observed equated to fully watered (FW) pots. Based on the relative SWC, the degree of drought stress was categorized as follows: control (CK), with 70–75% of fully watered content; mild drought (LD), with 50–55% of fully watered content; moderate drought (MD), with 30–35% of fully watered content; and severe drought (SD), with 10–15% of fully watered content. Each SWC treatment group comprised ten pots.

### 2.2. Volatile Metabolic Profiling Analysis

Basil leaves were ground into a powder using liquid nitrogen, and three replicates of each assay were conducted. The volatile compounds were analyzed by Wuhan Metware Biotechnology Co., Ltd. (Wuhan, China) on the Agilent 8890-7000D platform. For this analysis, 500 mg of each sample was weighed in a 20 mL headspace bottle with saturated NaCl solution. The samples were subjected to shaking at 60 °C for 5 min, and a 120 μm divinylbenzene/carboxen/polydimethylsiloxane fiber filter was exposed to the headspace to absorb volatiles for 15 min. Subsequently, GC-MS separation and identification were performed after analysis at 250 °C for 5 min.

Volatile organic compounds (VOCs) were identified and quantified using an 8890 GC and 7000 DMS system (Agilent, Santa Clara, CA, USA) equipped with a 30 m × 0.25 mm × 0.25 μm DB-5MS capillary column. Helium gas at 1.2 mL/minute served as the carrier gas and was programmed to begin at 40 °C (3.5 min); increase by 10 °C/min to 100 °C, 7 °C/min to 180 °C, and 25 °C/min to 280 °C; and maintain 280 °C for 5 min. Mass spectrometry (MS) employed a 70 eV electron impact ionization mode. Temperatures for the quadrupole mass detector, ion source, and transfer line were set at 150 °C, 230 °C, and 280 °C, respectively. To identify volatile compounds, mass spectra were compared with NIST data and the linear retention index.

### 2.3. Transcriptome Sequencing

Total RNA from basil leaves was isolated using plant RNA isolation kits (Thermo Fisher, Waltham, MA, USA). The RNA libraries for CK, LD, MD, and SD samples were prepared and sequenced, with three biological replicates conducted for each variety. The cDNA library was constructed and sequenced by Wuhan Metware Biotechnology Co., Ltd. (Wuhan, China).

After assessing RNA quality, mRNA was purified and fragmented and a cDNA library was created (with the Illumina NEBNext^®^ UltraTM RNA Library Prep Kit). The library was initially quantified with a qubit 2.0 fluorometer and diluted to 1.5 ng/µL, and its insert size was analyzed using an Agilent 2100 bioanalyzer. Once the library passed inspection, Illumina sequencing was conducted. The raw data were collected through Illumina paired-end sequencing on the Illumina HiSeq 4000 platform. Following the removal of poly-N and low-quality raw reads, the clean reads were assembled into expressed sequence tag clusters (contigs). De novo transcripts were constructed, and Q30 and GC contents were calculated using Trinity [35]. TransDecoder (https://github.com/TransDecoder/, accessed on 26 December 2024) was used to transcribe the Trinity assembly, predicting the amino acid sequence of the transcript. Htseq counts were employed to determine read counts for each unigenes and Cufflinks software (Cufflinks 2.x) was used to calculate fragments per kilobase million (FPKM). DIAMOND software (DIAMOND v2.x) was employed to align the deleted transcript sequences against the KEGG and HMMER software (HMMER 3.3.2) was utilized to compare amino acid sequences with the Pfam database.

### 2.4. Bioinformatics Analysis

To identify significantly regulated VOCs between each group, criteria of variable importance in projection (VIP) ≥ 1 and absolute Log2 (fold change) ≥ 1 were applied. A threshold of |log2 (fold change)| > 1 and a corrected *p*-value of less than 0.05 were used to screen for differentially expressed genes (DEGs). The screening process was facilitated by the MetaboAnalystR (MetaboAnalystR 2.0) package, extracting VIP values from Orthogonal Partial Least Squares Discriminant Analysis (OPLS-DA) results, which also encompassed score plots and permutation plots. Prior to OPLS-DA, the data underwent log transformation (log2) and mean centering. A total of 200 permutation tests were conducted to prevent overfitting.

Differentially expressed VOCs and DEGs were defined as described previously [36]. Unigenes underwent annotation with GO terms using the (http://www.geneontology.org, accessed on 1 March 2023) platform and were further analyzed with Blast2GO. The KEGG database was employed to annotate KEGG pathways, utilizing Blastall software (BLAST 2.x) for this purpose.

### 2.5. Assessment of Physiological and Biochemical Properties

The levels of malondialdehyde (MDA) were quantified through the thiobarbituric acid oxidation technique: the leaf extract was combined with 10% trichloroacetic acid and 0.65% thiobarbituric acid (TBA) and subsequently heated at 95 °C for 25 min. The absorbance was measured at OD532 and OD600; the available formula A532 − A600 = 155,000 × C × L was used to calculate the MDA concentration C (μmol/L), where L is the thickness of the colorimetric cup (cm).

The concentration of free proline was assessed using the ninhydrin colorimetric assay; proline was extracted from the plant samples using sulfosalicylic acid and then reacted with ninhydrin to form a red product under acidic conditions. The absorbance at 520 nm was measured and the proline content in the sample was determined using a standard curve.

H_2_O_2_ concentration in leaves was detected by the spectrophotometric analysis of titanium sulfate at 415 nm. For this, 0.3 g of ground leaves was mixed with 5 mL cold acetone, centrifuged at 3000× *g* for 10 min, and then mixed with 1 mL supernatant with 5% titanium sulfate and concentrated ammonia. After forming a precipitate, it was centrifuged for 3000 min, the supernatant was discarded, and the precipitate was washed 3–5 times with acetone to remove the pigment. Then, 5 mL of 2 M sulfuric acid was added to the cleaned precipitate and dissolved completely; the absorbance was measured at 415 nm.

The enzymatic activity of peroxidase, catalase, and superoxide dismutase was evaluated following established procedures [37]. To conduct the enzyme assays, 0.3 g of leaf material was ground with 3 mL of ice-cold 50 mM (including 0.2 mM EDTA and 2% PVP) PH7.8 phosphate buffer (PBS). The mixture was then centrifuged at 4 °C for 20 min at 12,000× *g*, and the supernatants obtained were used for further analysis. POD activity was assessed using guaiacol colorimetry by mixing a reaction liquid with enzyme liquid in a spectrophotometer cup and measuring the OD at 470 nm every minute. CAT activity was measured via ultraviolet spectrophotometry by adding enzyme solution to a reaction solution, using distilled water as a blank control, and recording the OD at 240 nm initially and every 30 s for six readings, repeated three times. SOD activity was determined using the NBT photoreduction method by reacting the supernatant with NBT solution under a specific light intensity and measuring the OD at 560 nm.

The α-linolenic acid content in basil leaves was assessed through GC-MS [38]. The leaves were dried and ground into a powder with liquid nitrogen. Hydrochloric acid, methanol, and petroleum ether were added in sequence to obtain the fat extract. Then, 1 mL of n-hexane was added for extraction over a period of 20 min. Subsequently, 1 mL of a 0.5 mol/L sodium methoxide solution was added for methyl-esterification, followed by vigorous shaking for 10 min. The mixture was filtered through a 0.22 μm pinhole filter membrane and transferred to a specialized sample vial for analysis. A quantitative analysis of α-linolenic acid was conducted using gas chromatography coupled with mass spectrometry (GC7890A/MS5975C, Agilent Technologies, Santa Clara, CA, USA), employing the internal standard curve method for quantification.

The quantification of flavonoid and flavonol contents in the samples was conducted using HPLC [39]. For this, 0.5 g of lyophilized leaves were extracted for 1 h at 50 °C in 50% aqueous methanol. After cooling, the extract was diluted to a final volume of 50 mL with methanol. Approximately 2 mL of this solution was filtered through a 0.22 μm filter, and 20 μL was injected into an Agilent 1260 high-performance liquid chromatograph for further analysis.

The endogenous lignin content was quantified employing a thioglycolic acid lignin method [40]. After drying, crushing, and sieving the basil leaves through a 60-mesh screen, they were accurately weighed to 5.0 mg and added to a 15 mL glass test tube. Then, 5 mL of 30% acetyl bromo-ice acetic acid solution and 200 μL of 1.0 mol/L perchloric acid were added and the tube was sealed. The mixture was heated in an 80 °C water bath for 40 min and then cooled. The solution was transferred to a 50 mL volumetric flask containing 10 mL each of 2 mol/L sodium hydroxide and glacial acetic acid to stop the reaction. It was mixed and diluted with glacial acetic acid to 50 mL. The product’s UV absorbance at 280 nm was measured, using an ice acetic acid solution without herbal powder as a blank control.

All data were analyzed and compiled using Excel 2010. Data variance was calculated utilizing SPSS27 software and statistical significance was assessed through Duncan’s multiple comparison method (*p* < 0.05).

### 2.6. Quantitative Real-Time PCR Validation

DEGs linked to α-linolenic and phenylpropionic acid pathways showing significant differences, high fpkm values, and complete CDS sequences were selected for qRT-PCR validation. Nine differentially expressed genes (DEGs) were selected to validate the results obtained from the RNA sequencing analysis. Complementary DNA (cDNA) was synthesized from the same RNA samples used in the transcriptome sequencing. The reverse transcription reaction was performed using the ReverTra Ace qPCR RT Master Mix (code no. FSQ-301) from TOYOBO (Toyo Boseki Kabushiki Kaisha, Osaka, Japan). The reaction mixture had a total volume of 10 μL, comprising 2 μL of 4x DN Master Mix, 2 μL of 5x RT Master Mix II, 1 μL of RNA template, and 5 μL of nuclease-free water, following the manufacturer’s protocol. Real-time quantitative PCR (qRT-PCR) was conducted using the iCycler iQ Multicolor Real-Time PCR Detection System from Bio-Rad, Hercules, CA, USA, in conjunction with the SYBR Green qRT-PCR Kit (code no. QKD-201) from TOYOBO, Osaka, Japan. The PCR reaction was prepared with a total volume of 20 μL, containing 10.0 μL of KOD SYBR^®^ qPCR mix, 1.0 μL of diluted cDNA, and 0.2 μM of each primer, with the remainder filled with double-distilled water. The cycling parameters were as follows: an initial denaturation at 98 °C for 2 min, followed by 40 cycles of denaturation at 98 °C for 10 s, annealing at 60 °C for 10 s, and extension at 68 °C for 30 s. Specific primers for gene amplification are shown in Supplementary Table S1. The candidate genes’ relative expression was quantified through the 2^−∆∆CT^ method.

## 3. Results

### 3.1. The Physiological and Biochemical Characteristics of Basil

**Figure 1:**
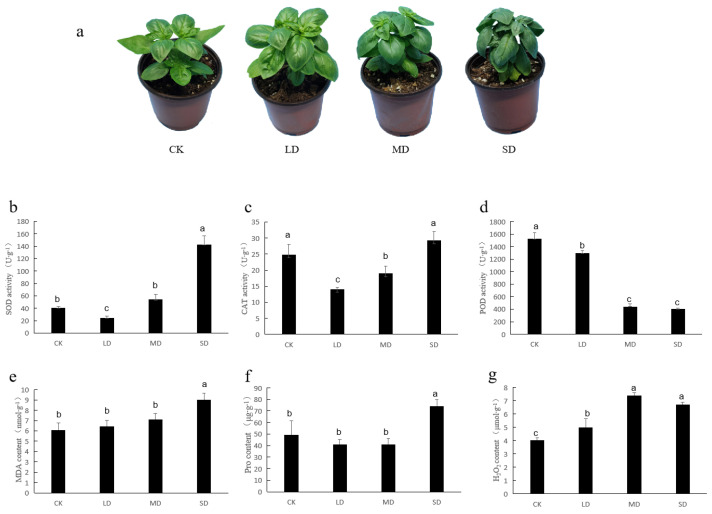
Variations in basil’s morphological, physiological, and biochemical traits occurred in response to varying degrees of drought stress: (**a**) phenotype; (**b**) SOD activity; (**c**) CAT activity; (**d**) POD activity; (**e**) MDA content; (**f**) proline content; (**g**) H_2_O_2_ content. Distinct lowercase letters signify that there were significant differences (*p* < 0.05) across various drought treatments for each corresponding index. CK stands for control, LD stands for mild drought, MD stands for moderate drought, SD stands for severe drought.

As the drought intensified, the basil leaves turned darker in color and wilted due to the loss of water (Figure 1a). At the same time, there was a change in the activity of antioxidant enzymes and the levels of osmoregulatory substances (Figure 1b–g). Compared with that of the CK, SOD activity decreased under LD treatment and increased under SD treatment; CAT activity decreased under LD and MD treatment; POD activity decreased under LD, MD, and SD stress; MDA and proline contents increased under SD stress; and H_2_O_2_ content increased under LD, MD, and SD stress; these differences were considerable (*p* < 0.05).

### 3.2. Volatile Metabolome Analysis of Sweet Basil Under Four Different Levels of Drought Stress

**Figure 2:**
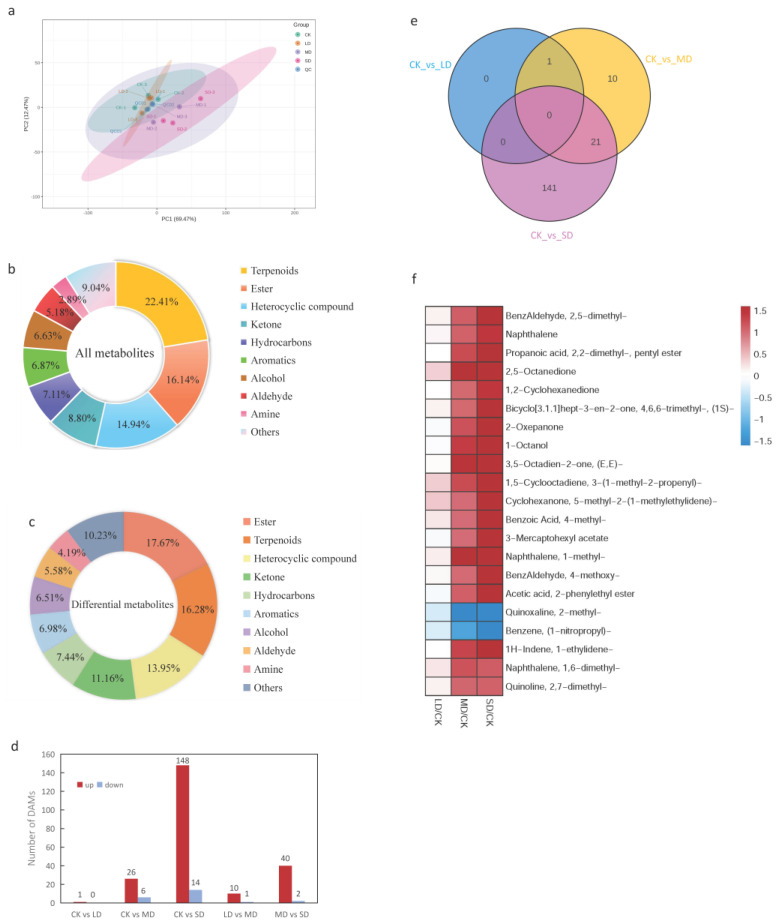
Analysis of metabolites. (**a**) Principal component analysis (PCA) between samples of different drought treatments. (**b**) Component analysis of the identified VOCs of sweet basil. (**c**) Component analysis of the identified differential VOCs of sweet basil under drought stress. (**d**) Histogram of upregulated and downregulated metabolites from paired comparisons. (**e**) Venn diagram of differential metabolites in a multiple pairwise comparison of CK vs. LD, CK vs. MD, and CK vs. SD. (**f**) Fold-changes (FCs) of 21 common differentially accumulated metabolites in the CK, MD, and SD groups. Blue indicates downregulated expression and red indicates upregulated expression. CK stands for control, LD stands for mild drought, MD stands for moderate drought, SD stands for severe drought, DAMs stands for differentially accumulated metabolites.

To explore the impact of drought stress on the VOCs in sweet basil, volatile metabolic profiling of sweet basil leaves was conducted under four gradient drought stress conditions. For the further elucidation of VOCs accumulating differentially in response to drought stress, LC-MS was utilized for volatile metabolic analysis. To analyze the dynamic changes of basil under drought stress, principal component analysis (PCA) was performed. As shown in Figure 2a, some basil samples under CK and LD treatments clustered together, while the separation of other samples indicated significant changes in metabolites.

OPLS-DA was employed to identify differential metabolites by extracting components of the independent variable X and dependent variable Y and calculating correlations between them. The results revealed R^2^X values higher than 0.93, R^2^Y scores higher than 0.99, and Q^2^ values higher than 0.52 in the CK vs. LD, LD vs. MD, and MD vs. SD samples, respectively. This confirmed the presence of differential metabolites responsive to drought (Supplementary Figure S1).

A total of 830 VOCs were identified (Supplementary Table S2), with linalool, eucalyptol, and eugenol representing a substantial proportion. The categorized VOCs included terpenoids (22.41%), esters (16.14%), heterocyclic compounds (14.94%), ketones (8.8%), hydrocarbons (7.11%), aromatics (6.87%), alcohols (6.63%), aldehydes (5.18%), amines (2.89%), and other compounds (9.04%), where terpedoids and esters collectively constituted 38.55% of the VOCs (Figure 2b). Metabolites with VIP values > 1 and *p* < 0.05 were considered significantly differentially accumulated. A total of 215 differentially accumulated metabolites were identified (Supplementary Table S3), categorized as esters (17.64%), terpenoids (16.28%), heterocyclic compounds (13.95%), ketones (11.16%), hydrocarbons (7.44%), aromatics (6.98%), alcohols (6.51%), aldehydes (5.58%), amines (4.19%), and other compounds (10.23%) (Figure 2c). A summary table was constructed to analyze the top 30 total and differential volatile compounds (Table 1). The analysis revealed that the concentrations of several key volatiles, such as eugenol and eucalyptol, remained relatively stable under drought stress conditions. Importantly, the differential volatiles were primarily ranked lower, with only the top five differential volatiles also appearing among the top 30 total volatiles (highlighted in yellow).

The metabolic results indicated 1 upregulated differential metabolite at CK vs. LD, 32 differential metabolites (26 up- and 6 downregulated) at CK vs. MD, 162 differential metabolites (148 up- and 14 downregulated) at CK vs. SD, 11 differential metabolites (10 up- and 1 downregulated) at LD vs. MD, and 42 differential metabolites (40 up- and 2 downregulated) at MD vs. SD (Figure 2d). Exploring the common differential metabolites in CK vs. LD, CK vs. MD, and CK vs. SD, a total of 173 differential metabolites were identified (Figure 2e). Focusing on CK vs. MD and CK vs. SD due to the minimal differences in CK vs. LD, 21 common metabolites were found in these two comparison groups (Supplementary Table S4).

To pinpoint drought stress-related metabolites in sweet basil leaves and discern their increasing/decreasing trends, 21 differentially accumulated metabolites common to drought stress and water sufficiency treatments were analyzed. Under drought stress, 19 metabolites exhibited an increase, while two metabolites displayed a decrease in both comparison groups. Notably, the contents of 2-methyl-quinoxaline and (1-nitropropyl)-benzene decreased gradually, while the rest of the metabolites increased continuously under drought stress. Minimal changes in metabolites were observed in the LD stage, but as it progressed to the MD and SD stages, the differences became stark (Figure 2f).

### 3.3. Transcriptome Analysis of Sweet Basil Under Four Different Gradients of Drought Stress

The sequencing analysis was conducted on the same materials used in the metabolomic analysis, yielding 86.63 Gb of clean data. Each sample reached 5 Gb, and the percentage of Q30 bases exceeded 93%. A total of 108,807 unigenes were assembled, with an N50 length of 2017 bp and an average GC ratio of 50.01% (Table 2). The sequencing data were of high quality and suitable for further analysis.

**Figure 3:**
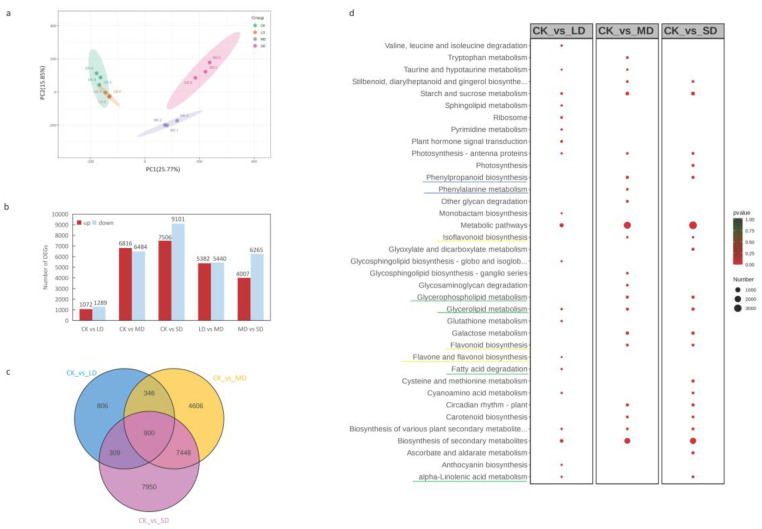
Transcriptome data analysis. (**a**) Principal component analysis (PCA) between samples of different drought treatments. (**b**) Histogram of upregulated and downregulated DEGs in different comparison groups. (**c**) Venn diagram of differential DEGs in a multiple pairwise comparison of CK vs. LD, CK vs. MD, and CK vs. SD. (**d**) Top 20 enriched KEGG pathways in different comparison groups of DEGs. CK stands for control, LD stands for mild drought, MD stands for moderate drought, SD stands for severe drought, DEGs stands for differentially expressed genes.

A PCA analysis of 12 samples revealed strong correlations between replicates within each group and weaker correlations between treatments (Figure 3a). The CK and MD treatment groups displayed the weakest correlation, while a good correlation was observed between CK and LD. A total of 27,181 differentially expressed DEGs were identified in this study. A comparison of upregulated and downregulated DEGs in different drought treatments is illustrated in Figure 3b. For LD/CK, there were 2361 DEGs (1072 upregulated and 1289 downregulated); for MD/CK, 13,300 DEGs (6816 upregulated and 6484 downregulated); for SD/CK, 16,607 DEGs (7506 upregulated and 9101 downregulated); for MD/LD, 10,822 DEGs (5382 upregulated and 5440 downregulated); and for SD/MD, 10,272 DEGs (4007 upregulated and 6265 downregulated). The Venn diagram demonstrates that among all three treatment groups, 900 common DEGs were differentially expressed (Figure 3c), suggesting their involvement in the response to drought stress.

To unravel the biological functions of DEGs, the top 20 enriched KEGG pathways among the groups LD/CK, MD/CK, and SD/CK were screened (Figure 3d, Supplementary Table S5). Metabolic pathways emerged as the most significant co-enrichment pathways. Glucose metabolism, such as starch and sucrose metabolism, and lipid metabolism (underline green), like glycerolipid metabolism, contained a substantial number of DEGs enriched in all three comparison groups. Phenylpropane (Underline blue) and flavonoid (underline yellow) biosyntheses also made up a considerable proportion.

### 3.4. Verification of Metabolite Levels

**Figure 4:**
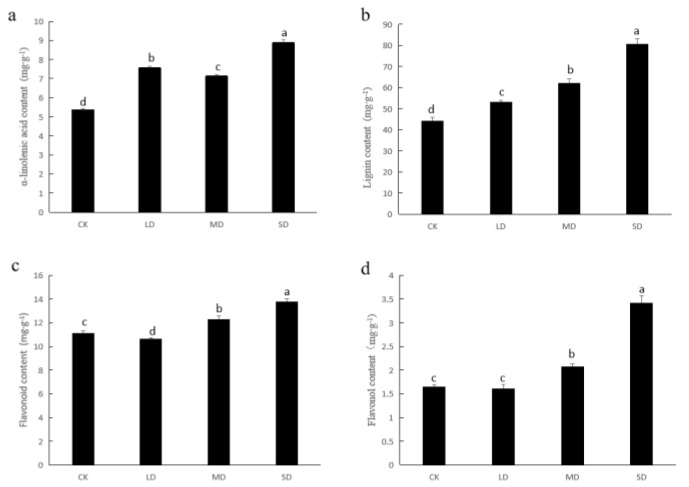
Verification of metabolites content: (**a**) α-linolenic acid content; (**b**) lignin content; (**c**) flavonoid content; (**d**) flavonol content. The different letters indicate significant differences (*p* < 0.05). CK stands for control, LD stands for mild drought, MD stands for moderate drought, SD stands for severe drought.

Owing to the poor conjunction between metabolites and transcriptome outcomes, we concentrated mainly on analyzing stress-related aspects of the transcriptome. According to KEGG enrichment analysis of the transcriptome data, it was discovered that lipid metabolism and phenylpropanoid pathways appear to play a significant role in the response of basil to drought stress. Therefore, we selected α-linolenic acid, lignin, flavonoid, and flavonol contents for analysis to substantiate our hypothesis. Interestingly, the levels of these four substances rose considerably during drought stress. In comparison to CK, α-linolenic acid and lignin contents demonstrated a significant increase under all three levels of drought stress. The flavonoid and flavonol contents exhibited a significant increase in MD and SD stress (Figure 4).

### 3.5. Responses of Key Genes to α-Linolenic Acid and Phenylpropanoid Pathways

**Figure 5:**
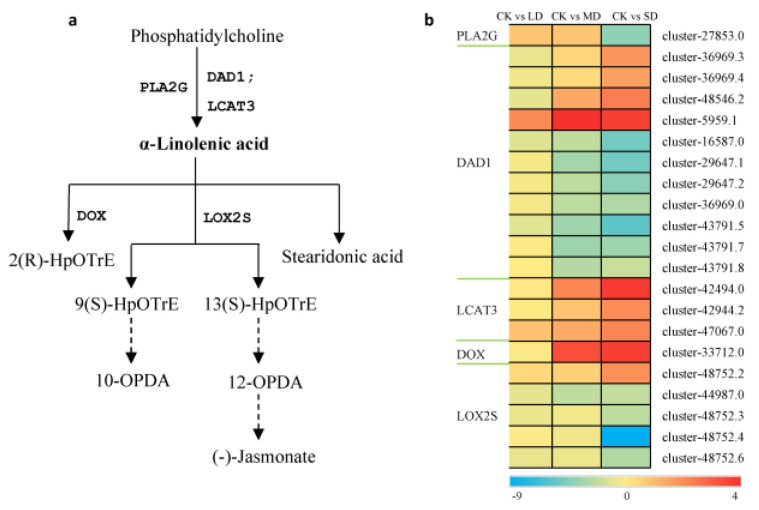
Differential expression of DEGs related to the linolenic acid metabolic pathway. Log2FC ratio was used to represent the differential changes in DEG content. Blue represents a decrease in content and red represents an increase. CK stands for control, LD stands for mild drought, MD stands for moderate drought, SD stands for severe drought. DAD1, phospholipase A1; LCAT3, phospholipase A1; PLA2G, secretory phospholipase A2; LOX2S, lipoxygenase; DOX, fatty acid alpha-dioxygenase.

The results above revealed active responses in the α-linolenic acid and phenylpropanoid pathways to drought stress. We show the synthesis and metabolism pathway map of α-linolenic acid (Figure 5a), as depicted in Figure 5, 21 unigenes were identified in the α-linolenic acid metabolic pathway (Figure 5b). *PLA2G*, *DAD1*, and *LCAT3* were identified as the three key genes synthesizing α-linolenic acid. As drought intensity increased, the expression of *PLA2G* and most of the *DAD1* unigenes decreased, while all *LCAT3* unigenes were upregulated. Downstream of α-linolenic acid, the expression of *DOX* was upregulated, while most *LOX2S* were downregulated.

**Figure 6:**
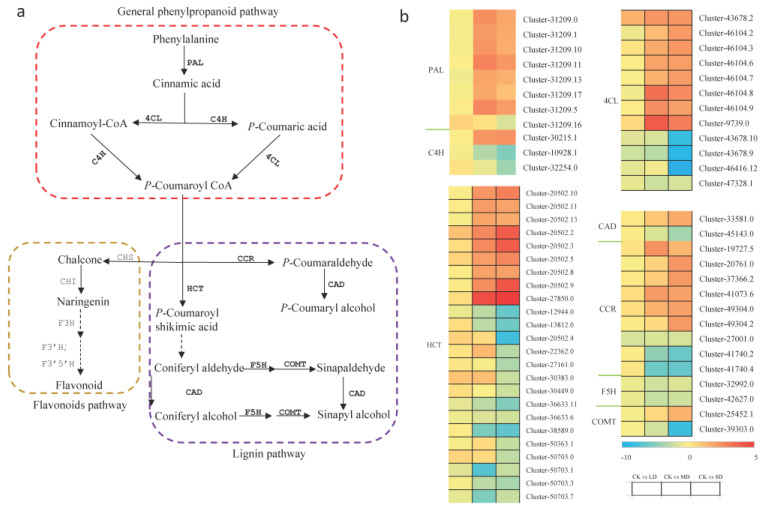
Differential expression of DEGs related to the phenylpropanoid, flavonoid, and lignin pathways. Log2FC ratio was used to represent the differential changes in DEG content. Blue represents a decrease in content and red represents an increase. The DEGs written in grey were not differentially expressed in this study. CK stands for control, LD stands for mild drought, MD stands for moderate drought, SD stands for severe drought. PAL, phenylalanine ammonia lyase; C4H, cinnamate 4-hydroxylase; 4CL, 4-coumaroyl CoA ligase; CHS, chalcone synthase; CHI, chalcone isomerase; F3H, flavanone 3 β-hydroxylase; F3’H, flavonoid 3’-hydoxylase; F3’5’H, flavonoid 3’,5’-hydoxylase; HCT, hydroxycinnamoyl-CoA:shikimate/quinate hydroxycinnamoyl transferase; F5H, ferulate 5-hydroxylase; COMT, caffeic acid O-methyltransferase; CAD, cinnamyl alcohol dehydrogenase.

Regarding the phenylpropanoid pathway, which is associated with the flavonoid and lignin pathways (Figure 6a), it encompassed 62 differentially expressed unigenes (Figure 6b). The majority of *PAL* and *4CL* unigenes were upregulated, while two-thirds of *C4H* unigenes were downregulated. *HCT*, a large family containing 24 differentially expressed unigenes, showed nine upregulated, six downregulated, and nine upregulated in MD/CK and downregulated in SD/CK. *CCR*, containing nine differentially expressed unigenes, displayed six upregulated and three downregulated. *CAD*, *F5H*, and *COMT*, essential genes for lignin synthesis, had two downregulated unigenes for *F5H*, while *CAD* and *COMT* had two differentially expressed unigenes, with one upregulated and one downregulated.

### 3.6. Verification of Differential Gene Expression Using Quantitative Real-Time RT-PCR

**Figure 7:**
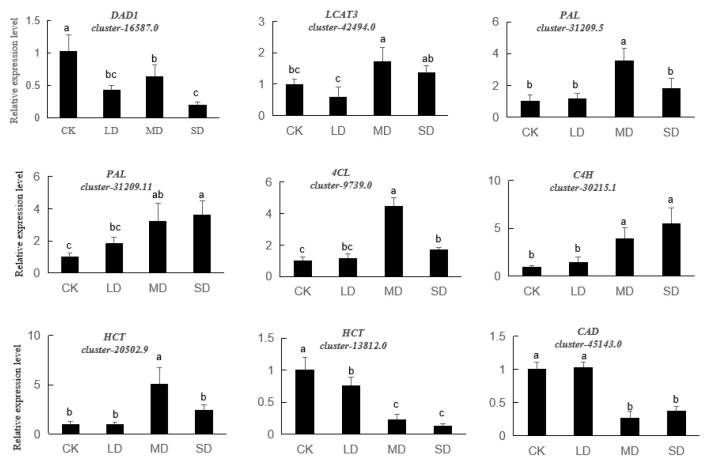
qRT-PCR analysis of nine candidate unigenes related to drought stress. The different letters indicate significant differences (*p* < 0.05). CK stands for control, LD stands for mild drought, MD stands for moderate drought, SD stands for severe drought.

The expression of nine DEGs in sweet basil was validated using qRT-PCR (Figure 7), with two genes linked to the linolenic acid metabolic pathway and seven genes tied to the phenylpropanoid pathway. Compared with that of the CK, the relative expression of *PAL*, *4CL*, *C4H*, and *HCT* (cluster20502.9) increased significantly, while the relative expression of *DAD1*, *HCT* (cluster13812.0), and *CAD* decreased significantly and *LCAT3* decreased under LD treatment and increased under SD treatment. These results were in agreement with the transcriptomic data.

## 4. Discussion

**Figure 8:**
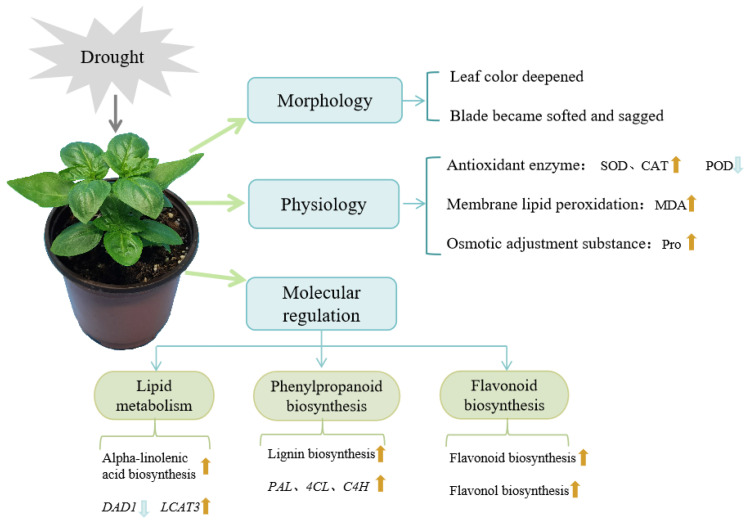
Extensive analysis of sweet basil’s adaptation to drought stress. SOD, superoxide dismutase; CAT, catalase; POD, peroxidase; MDA, malondialdehyde; Pro, proline; DAD1, phospholipase A1; LCAT3, phospholipase A1; PAL, phenylalanine ammonia lyase; C4H, cinnamate 4-hydroxylase; 4CL, 4-coumaroyl CoA ligase.

*Ocimum basilicum* L. is an annual medicinal aromatic plant renowned for its essential oil, which holds significant economic value. While basil exhibits good drought tolerance, prolonged drought stress can result in diminished plant yield, reduced quality, and even plant mortality. Therefore, investigating the drought response mechanism of basil is of paramount importance. In this study, an analysis of the morphological characteristics, volatile metabolome, and transcriptome data of sweet basil under varying degrees of drought stress was conducted. These observations uncovered the physiological and molecular responses of sweet basil to drought stress, revealing pronounced differences in the accumulation of metabolites, signal transmission, and gene expression (Figure 8).

The impact of drought encompasses various physiological and biochemical processes, which include photosynthesis, the antioxidant system, osmotic regulation, membrane lipid peroxidation, and so on [41]. In this study, the activities of SOD and CAT, along with the levels of MDA and proline, were found to increase in response to drought stress (Figure 1). This suggests that basil combats drought stress by enhancing the activity of antioxidant enzymes, eliminating reactive oxygen species, and accumulating osmoregulatory substances. Sweet basil leaves were found to contain 830 volatile metabolites using the SPME-GC-MS method. The primary components of VOCs included terpenoids, esters, heterocyclic compounds, hydrocarbons, aromatics, alcohols, aldehydes, ketones, and other compounds. Notably, linalool, eucalyptol, and eugenol were the main components (Table 1). These findings align with reports on the essential oil composition in different basil species [22]. A total of 173 differentially expressed VOCs were identified among the common differential metabolites in CK vs. LD, CK vs. MD, and CK vs. SD. However, with the exception of benzaldehyde, the content of major components such as linalool, cineole, and others exhibited no significant changes, indicating that the primary volatiles of basil remain relatively stable under drought stress. This stability has been corroborated by postharvest drying experiments with basil [42]. Twenty-one common metabolites were identified in CK vs. MD and CK vs. SD comparison groups, among which, 19 metabolites were upregulated and only two substances were downregulated. With the increasing severity of drought stress, the content of benzene and quinoxaline decreased, suggesting their potential participation in the synthesis of other metabolites as substrates.

In this study, the top 20 enriched KEGG pathways of DEGs demonstrated that both primary and secondary metabolism exhibited positive responses to drought stress (Figure 3d). The enriched pathways encompassed glucose metabolism, lipid metabolism, photosynthesis, secondary metabolite synthesis, and others. This pattern mirrored the KEGG enrichment pathways observed in other plants under drought stress, such as maize [43], millet [44], soybean [45], and rice [46]. Intriguingly, the different DEGs were mainly enriched in phenylpropyl and flavonoid biosynthesis and lipid metabolism. These pathways likely play a crucial role in the response of basil plants to drought stress.

A plant’s metabolism plays a pivotal role in its resistance to stress. The accumulation of specialized metabolites, including flavonoids, terpenes, and phenols, is known to be significant across various plant species [47,48,49,50]. Flavonoids and lignins are important for plant growth and self-protection from extreme environmental conditions. Previous studies have highlighted the importance of fatty acid metabolism and the phenylalanine metabolism pathway in drought stress responses [51,52,53,54]. Fatty acids, which make up the cell membranes of plants, play an important role in signal transmission and metabolism. As the precursor of flavonoid and lignin metabolisms, the plant phenylalanine metabolism has always been considered an important secondary metabolic pathway. Despite the transcriptome–metabolome association results not meeting expectations, our biochemical and transcriptomic analyses successfully identified two crucial metabolic pathways in basil that respond to drought stress: the α-linolenic acid and phenylalanine metabolic pathways (Figure 5 and Figure 6).

In the α-linolenic acid metabolic pathway, 21 unigenes exhibited significant changes in gene expression. Among them, cluster-5959.1, cluster-42494.0, and cluster-33712.0 showed the most noticeable upregulation of expression, while cluster-48752.4 showed the most evident downregulation of expression. These clusters may be key genes in response to drought stress in α-linolenic acid metabolic pathways. Downstream of α-linolenic acid, the expression of DOX was upregulated markedly, while most LOX2S genes were downregulated, suggesting that α-linolenic acid was less involved in the jasmonic acid pathway as a substrate. Therefore, the content of α-linolenic acid increased under drought stress (Figure 6), which differed from a previous report that found a decrease in linolenic acid content under drought stress [51]. This discrepancy might also be attributable to differences in plant species and the degree of drought.

The phenylpropanoid metabolic pathway serves as a crucial route from which various secondary metabolites, such as flavonoids and lignins, originate. In the present study, a total of 62 differentially expressed unigenes were encompassed in the phenylpropanoid and lignin pathways and the majority of *PAL* unigenes were upregulated. As a key gene in phenylpropanoid metabolism, it may promote downstream substrate synthesis. Meanwhile, when the soil was relatively dry (MD and SD), the flavonoid, flavonol, and lignin contents all increased significantly (Figure 7). These findings suggest that the phenylpropanoid metabolic pathway may be a key regulatory pathway in the response of sweet basil to drought stress.

In this study, an integrated network of volatile metabolites and transcriptome is proposed based on omics data. The changes in nonvolatile metabolites involved in the sweet basil’s response to drought stress, however, remain unclear. In addition, more research is needed to determine how structural genes and transcription factors influence basil’s drought tolerance. Through these, we will gain a better understanding of basil’s drought stress response at the molecular level.

## 5. Conclusions

In this study, the volatile metabolome and transcriptome of sweet basil were systematically analyzed under various degrees of drought stress. Basil developed typically under CK and LD conditions, but faced stress under MD and SD conditions, leading to a significant rise in malondialdehyde (MDA), hydrogen peroxide (H_2_O_2_), and proline levels and superoxide dismutase (SOD) activity and a notable reduction in the activities of peroxidase (POD) and catalase (CAT). In total, 830 metabolites were meticulously identified, of which 215 were differentially accumulated. The primary components of sweet basil volatiles remained stable under the influence of drought stress. Significant enrichment was observed in the DEGs associated with phenylpropanoid and flavonoid biosynthesis and lipid metabolism. α-linolenic acid, lignin, flavonoid, and flavonol contents significantly increased under stress; the essential genes involved in the production of these compounds were confirmed through quantitative real-time PCR (qRT-PCR) and their variations aligned with the outcomes from sequencing. These findings provide valuable insights for screening drought-tolerant basil varieties and advancing the molecular breeding of basil with enhanced drought tolerance.

## Figures and Tables

**Table 1:** Comparison between the top 30 total metabolites and distinct metabolites.

All Metabolites			Significantly Different Metabolites		
Index	Compounds	Class I	Index	Compounds	Class I
KMW0540	eugenol	Phenol	NMW0155	propofol	Phenol
KMW0544	methyleugenol	Phenol	KMW0519	vanillin	Aldehyde
KMW0218	eucalyptol	Terpenoids	KMW0235	1,3,6-octatriene, 3,7-dimethyl-, *(Z)-*	Terpenoids
XMW1333	benzoic acid, 3,4-dimethyl-, methyl ester	Ester	XMW0726	benzene, (1-methoxypropyl)-	Aromatics
XMW1256	2-butanone, 4-(2,6,6-trimethyl-1,3-cyclohexadien-1-yl)-	Ketone	NMW0177	*N*-benzylformamide	Amine
XMW0690	cyclohexene, 2,4-dimethyl-1-(1-methylethenyl)-	Hydrocarbons	XMW0742		Terpenoids
KMW0291	linalool	Terpenoids	KMW0212	benzeneacetaldehyde	Aldehyde
w41	3-octen-2-one, (*E*)-	Ketone	XMW1077	1,3-dioxolane-2,2-diethanol	Alcohol
XMW0285	benzaldehyde, 4-hydroxy-	Aldehyde	XMW0416	1,3-cyclohexadiene, 5-butyl-	Hydrocarbons
XMW0468	bicyclo[3.1.1]hept-2-ene, 2,6-dimethyl-6-(4-methyl-3-pentenyl)-	Terpenoids	NMW0021	2-pyridinemethanamine	Heterocyclic compound
NMW0158	benzene, 1-methoxy-2-nitro-	Aromatics	D99	benzoic acid, 2-(dimethylamino)-, methyl ester	Ester
NMW0156	benzene, (isothiocyanatomethyl)-	Sulfur compounds	KMW0679	2-propenoic acid, 3-phenyl-, methyl ester, *(E)-*	Ester
XMW0267	2-ethylpiperidine	Heterocyclic compound	KMW0178	bicyclo[3.1.0]hexane, 4-methylene-1-(1-methylethyl)-	Terpenoids
KMW0247	β-phellandrene	Terpenoids	D152	propanoic acid, 2-methyl-, 2-phenylethyl ester	Ester
XMW1209	propanoic acid, 2-methyl-, 2-methylbutyl ester	Ester	NMW0038	benzenemethanol, 4-methyl-	Alcohol
NMW0176	benzeneacetamide	Amine	XMW0076	1H-Pyrrole-3-carbonitrile	Heterocyclic compound
XMW1124	2-azabicyclo[3.2.1]octan-3-one	Ketone	XMW0244	2-butanone, 4-(2,6,6-trimethyl-1-cyclohexen-1-yl)-	Ketone
NMW0155	propofol	Phenol	XMW0142	3a,7-methano-3aH-cyclopentacyclooctene, 1,4,5,6,7,8,9,9a-octahydro-1,1,7-trimethyl-, [3aR-(3a.alpha.,7.alpha.,9a.beta.)]-	Terpenoids
XMW0119	cyclopentanone, 2-methyl-3-(1-methylethyl)-	Ketone	KMW0258	2-methoxy-phenol	Phenol
KMW0329	camphor	Terpenoids	KMW0267	benzoic acid, methyl ester	Ester
KMW0519	vanillin	Aldehyde	XMW0698	1,4-methano-1H-indene, octahydro-4-methyl-8-methylene-7-(1-methylethyl)-, [1S-(1.alpha.,3a.beta.,4.alpha.,7.alpha.,7a.beta.)]-	Terpenoids
KMW0235	1,3,6-octatriene, 3,7-dimethyl-, *(Z)-*	Terpenoids	w39	2-butanone, 4-(2,6,6-trimethyl-2-cyclohexen-1-yl)-	Ketone
XMW0909	2,6,6-trimethylbicyclo[3.2.0]hept-2-en-7-one	Ketone	XMW0023	4-methyl-1-(1-methylethyl)-bicyclo[3.1.0]hex-2-ene	Hydrocarbons
XMW0535	*p*-tolylacetic acid	Acid	KMW0278	Ethanone, 1-(2-thienyl)-	Heterocyclic compound
XMW0726	benzene, (1-methoxypropyl)-	Aromatics	XMW0548	bicyclo[7.2.0]undecane, 10,10-dimethyl-2,6-bis(methylene)-, [1S-(1R*,9S*)]-	Terpenoids
XMW1227		Ester	KMW0193	β-pinene	Terpenoids
XMW0228	cyclohexanol, 1-ethenyl-	Alcohol	D156	propanoic acid, 2-methyl-, 3-phenylpropyl ester	Ester
XMW0410	1-nonen-4-ol	Alcohol	NMW0185	pyridine, 3-(3,4-dihydro-2H-pyrrol-5-yl)-	Heterocyclic compound
KMW0217	D-limonene	Terpenoids	NMW0173	2,6-dihydroxy-benzoic acid	Acid
NMW0177	*N*-benzylformamide	Amine	KMW0148	α-pinene	Terpenoids

**Table 2:** Summary of RNA sequencing and assembly.

Sample	Raw Reads	Clean Reads	Clean Base (G)	Error Rate (%)	Q20 (%)	Q30 (%)	GC Content (%)
CK-1	52,927,868	507,06,010	7.61	0.03	98.03	94.32	49.39
CK-2	50,255,254	48,223,864	7.23	0.03	97.56	93.3	48.98
CK-3	44,466,932	42,657,780	6.4	0.03	97.62	93.44	49.77
LD-1	50,577,948	49,032,022	7.35	0.03	97.51	93.2	50
LD-2	45,867,542	44,270,194	6.64	0.03	97.56	93.32	50.02
LD-3	49,304,820	47,517,542	7.13	0.03	98.1	94.51	50.63
MD-1	46,598,338	45,007,078	6.75	0.03	97.63	93.42	50.15
MD-2	47,733,680	46,422,380	6.96	0.03	97.56	93.3	50.12
MD-3	58,463,114	57,043,684	8.56	0.03	98	93.99	49.48
SD-1	42,059,900	40,944,928	6.14	0.03	97.97	93.9	50.61
SD-2	40,507,526	38,994,948	5.85	0.03	98.1	94.28	50.25
SD-3	68,874,522	66,712,058	10.01	0.03	98.05	94.11	50.73

CK-1, CK-2, CK-3 stands for the three replicates of the control; LD-1, LD-2, LD-3 stands for the three replicates of mild drought; MD-1, MD-2, MD-3 stands for the three replicates of moderate drought; SD-1. SD-2, SD-3 stands for the three replicates of severe drought.

## Data Availability

The datasets generated and analyzed during the current study are available from the corresponding author upon reasonable request.

## Supplementary Materials

The following supporting information can be downloaded at https://www.mdpi.com/article/10.3390/cimb47020117/s1.
