# Supplementary material for: Analysis of Volatile Metabolome and Transcriptome in Sweet Basil Under Drought Stress
Source: Curr Issues Mol Biol. 2025 Feb 11;47(2):117. doi: 10.3390/cimb47020117 (PMC11854773; doi:10.3390/cimb47020117)
Supplement: Supplementary file 1 [file cimb-47-00117-s001.zip › supplementary Figure S1.pdf]

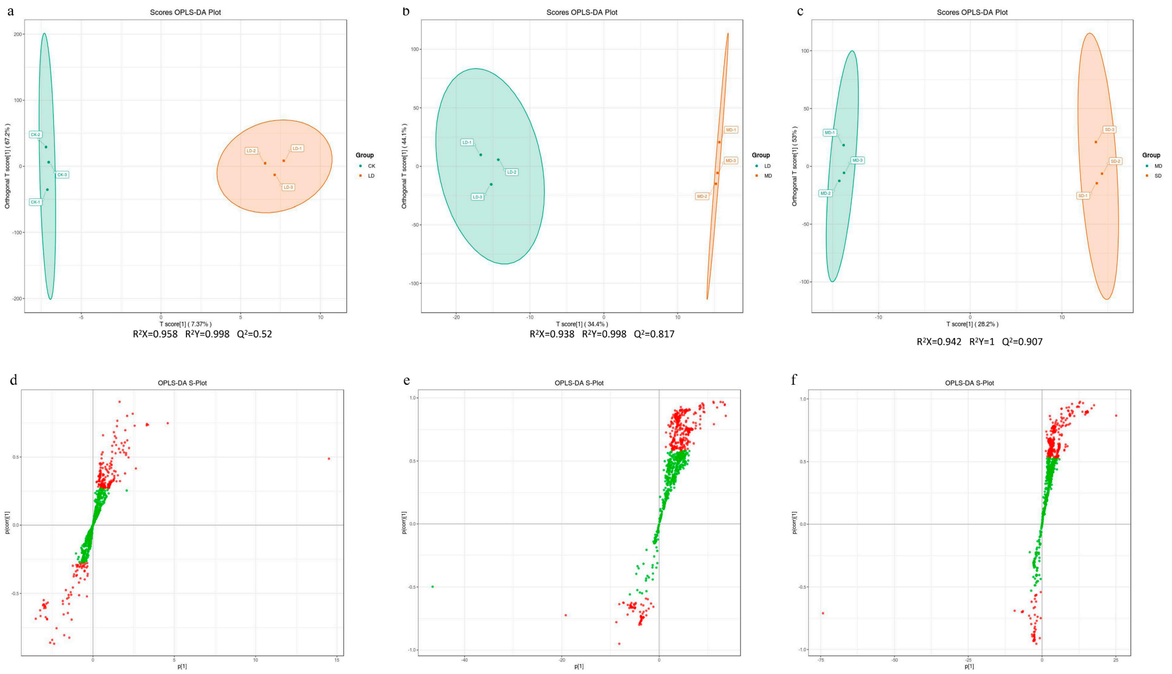

Supplementary Figure S1. Orthogonal partial least squares-discriminant analysis (OPLS-DA) scores. Scores of the OPLS-DA model, in order from left to right, CK vs LD, LD vs MD, MD vs SD; (d-f) PLS-DA S-plot model of CK vs LD, LD vs MS and MS vs SD in turn.
